# Prognostic Value of CT Radiomic Features in Resectable Pancreatic Ductal Adenocarcinoma

**DOI:** 10.1038/s41598-019-41728-7

**Published:** 2019-04-01

**Authors:** Farzad Khalvati, Yucheng Zhang, Sameer Baig, Edrise M. Lobo-Mueller, Paul Karanicolas, Steven Gallinger, Masoom A. Haider

**Affiliations:** 1 Department of Medical Imaging, University of Toronto, Toronto, ON Canada; 2 Lunenfeld-Tanenbaum Research Institute, Sinai Health System, Toronto, ON Canada; 3 Cross Cancer Institute, University of Alberta, Edmonton, AB Canada; 4 Department of Surgery, Sunnybrook Health Sciences Centre, University of Toronto, Toronto, ON Canada; 5 PanCuRx Translational Research Initiative, Ontario Institute for Cancer Research, Toronto, ON Canada; 6 Hepatobiliary Pancreatic Surgical Oncology Program, University Health Network, Toronto, ON Canada; 7 Sunnybrook Research Institute, Sunnybrook Health Sciences Centre, Toronto, ON Canada

## Abstract

In this work, we assess the reproducibility and prognostic value of CT-derived radiomic features for resectable pancreatic ductal adenocarcinoma (PDAC). Two radiologists contoured tumour regions on pre-operative CT of two cohorts from two institutions undergoing curative-intent surgical resection for PDAC. The first (n = 30) and second cohorts (n = 68) were used for training and validation of proposed prognostic model for overall survival (OS), respectively. Radiomic features were extracted using PyRadiomics library and those with weak inter-reader reproducibility were excluded. Through Cox regression models, significant features were identified in the training cohort and retested in the validation cohort. Significant features were then fused via Cox regression to build a single radiomic signature in the training cohort, which was validated across readers in the validation cohort. Two radiomic features derived from Sum Entropy and Cluster Tendency features were both robust to inter-reader reproducibility and prognostic of OS across cohorts and readers. The radiomic signature showed prognostic value for OS in the validation cohort with hazard ratios of 1.56 (P = 0.005) and 1.35 (P = 0.022), for the first and second reader, respectively. CT-based radiomic features were shown to be prognostic in patients with resectable PDAC. These features may help stratify patients for neoadjuvant or alternative therapies.

## Introduction

Pancreatic ductal adenocarcinoma (PDAC) is the third most common cause of cancer-related death in the US with an extremely poor prognosis. Over the past four decades, the 5-year survival rate has only marginally increased from 3% to 8.5%^1^. As the only definitive treatment, about 20% of PDAC cases are eligible for surgical resection^2^ with these patients having a 5-year survival of 19%^3^.

The most common clinicopathologic factors significantly associated with 5-year survival are lymph node status, tumour size, margin status at surgery, histological tumour grade, and receipt of adjuvant chemotherapy^3–6^.

As an evolving paradigm in cancer biomarker discovery and validation, *radiomics* has shown early promise in exploiting the latent information in medical images and establishing links between quantitative imaging biomarkers, and patient outcome and response to systemic chemotherapy and radiation^7–9^. Radiomics refers to the extraction and analysis of a large amount of quantitative features from medical images^10,11^. These quantitative imaging features can be used to build prognostic models to risk-stratify patients based on different clinical outcomes such as survival. The ability to capture the entirety of a tumour gives radiomics the capability of assessing one of the key features of cancer, heterogeneity. Radiomic parameters related to heterogeneity have been shown to be a prognostic factor for patient outcome in other cancer sites such as lung^12^.

Radiomic features include different classes of quantitative imaging features that each captures a different property of a region of interest (ROI). Fist-order (e.g., intensity) and second-order statistical features (e.g., texture such as contrast and homogeneity) are among the most frequently used radiomic features where the former are calculated using the histogram of grey-level pixels, regardless of the spatial relationship among the pixels, and the latter are calculated using grey level co-occurrence matrices (GLCM)^13^. Other radiomic feature classes include morphological features to capture ROI shape characteristics^14^ and edge detection features that highlight the boundaries of objects in the ROI^15^.

In PDAC, computed tomography (CT) is the main diagnostic tool for assessment of local extent of disease and surgical planning^16^. As a standard-of-care imaging modality, CT images can be used to extract radiomic features with no extra image acquisition cost to the healthcare system, thus providing comprehensive information on the phenotypic and textural structure of the tumour. Neoadjuvant therapy has been shown to improve the survival of patients with resectable PDAC^17^. If radiomic features can identify patients with more aggressive disease, it might help select patients most in need of neoadjuvant treatment.

Although previous studies have shown the prognostic value of CT radiomic features for different cancer sites including non-small cell lung cancer^7,18^, renal clear cell carcinoma^19,20^, and metastatic colorectal cancer^21^, there is scarcity of multicentre radiomics research on PDAC. Our preliminary results were published on a single small cohort (n = 30) exploring a limited number of radiomic features for prognostication (5 second-order statistical features) showing two features were predictors of overall survival (OS) for PDAC patients undergoing curative intent surgical resection^22^. Another study investigated radiation induced changes in CT radiomic features (8 first-order features) in a cohort of 20 patients with pancreatic head cancer over a period of 5 weeks and association between changes in radiomic features and pathologic response was reported^9^. Recently, a larger cohort of 161 patients with resected PDAC was analyzed to study the prognostic accuracy of radiomic features combined with preoperative serum carbohydrate antigen 19-9 (CA19-9 levels) and pathology score (The Brennan score) where it was reported that adding the pathology score to radiomic features and serum cancer antigen improves the prognostic power of the model^23^.

In this retrospective study, we aimed to address the shortcomings of the previous radiomic studies of PDAC by including assessment of reproducibility across different readers, using data from different institutions and CT scanners, and using separate training and validation sets. To achieve this goal and further validate CT radiomic parameters as prognostic biomarkers in PDAC patients, we investigated these parameters in two separate pre-operative cohorts from two institutions and contoured by two radiologists with different levels of expertise through the analysis of a comprehensive set of radiomic features with a standard analytic library (PyRadiomics version 2.0.1)^24^. The purpose of this study was to assess the reproducibility and prognostic value of CT-derived radiomic features for resectable PDAC.

## Materials and Methods

### Patients

This retrospective study was approved by the Research Ethics Board of Sunnybrook Health Sciences Centre and University Health Network and all methods were carried out in accordance with relevant guidelines and regulations. Two cohorts from two separate institutions consisting of 30 and 68 patients undergoing curative intent surgical resection for PDAC from 2007–2012 and 2008–2013, respectively, who had pre-operative contrast-enhanced CT available for analysis and were part of ongoing studies where survival data was being collected were included. Patients were resectable and had not received neo-adjuvant treatment. To minimize the effect of post-operative complications on outcomes analyses, patients who died within 90 days after surgery were excluded. Institutional review board approval was obtained for this study from both institutions and the need for written informed patient consent was waived. The demographic information for both cohorts is shown in Table 1.

**Table 1:** Cohorts’ demographic information.

		Cohort 1	Cohort 2
Age (years)	Mean ± Standard Deviation	69 ± 8	65 ± 11
Sex	Male/Female/Total	13/17/30	35/33/68
Size (diameter - cm)	Mean ± Standard Deviation	3.76 ± 0.97	4.34 ± 1.47
Grade	G1/G2/G3/G4/Total	3/19/8/0/30	17/44/6/1/68
Patients with Negative/Positive Nodes (N stage)	N0/N1/Total	6/24/30	15/53/68
Margin	R2/R1/R0/Total	0/16/14/30	0/10/58/68
CA19-9 (U/ml)	Mean ± Standard Deviation	893 ± 1514	2241 ± 9118
Survival Time (months)	Mean ± Standard Deviation	31 ± 25	25 ± 16

We previously used the first cohort (n = 30) in a pilot study where only few in-house developed radiomic features extracted from single reader contours were investigated for prognostic value of OS in PDAC patients^22^.

### Image acquisition

Patients underwent contrast-enhanced CT with a biphasic pancreas protocol consisting of arterial or pancreatic phase and portal venous phase acquisitions. As CT scans were not all from the same institution, the exact contrast bolus volume, timing, and injection rate varied over the time period. In addition, there was inconsistent timing related to variation in CT protocols during the arterial/pancreatic phase imaging. This resulted in variable enhancement of the tumour and background pancreas. As a result, in many cases, the tumour was inconsistently visualized on the arterial/pancreatic phase. The portal phase was consistent in timing and enhancement of background tissue across the entire cohorts. For these reasons, all pancreatic cancer boundaries were drawn on the portal venous phase of acquisition as this phase was most consistent across all exams. CT images were reconstructed with 5 mm and 2 mm intervals for the first cohort and second cohort, respectively. Detector width was 40 mm and kV was 120 kVp for the portal phase for both cohorts. Examination was performed on a 64 row multidetector helical CT (first cohort: GE Medical Systems, LightSpeed VCT, second cohort: Toshiba, Aquilion).

### Image analysis

An in-house developed volume of region contouring tool (ProCanVAS)^25^ was used by an experienced radiologist with 18 years of experience as an oncologic imager (Reader 1) and a radiology research fellow (Reader 2) blinded to patient outcome, to review the images and contour the ROIs on the slice with the largest visible cross section of the tumour on the portal venous phase. To differentiate the tumour and the pancreas background, relative contrast difference was considered and in cases where tumour boundary was not clear, tumour boundary was defined by the presences of pancreatic or common bile duct cut-off and review of pancreatic phase images^22^.

Feature extraction was performed on the ROI using the PyRadiomics library (version 2.0.1) in Python^24^. To remove the fat and stents, the images were thresholded where voxels with HU (Hounsfield unit) <−10 and >500 were excluded from the analysis. We used a subset of well-known PyRadiomics features, which include first order features and second order features extracted from GLCM matrix using different filters (no filter, exponential, gradient, logarithm, square, square-root, and local binary pattern filters). In total, 410 radiomic features were extracted which included different classes of features listed in Table 2.

**Table 2:** List of radiomic feature classes and filters.

First-order features	Histogram-based features
Second-order texture features	Features extracted from Gray-Level Co-Occurrence matrix (GLCM)
Morphology features	Features based on the shape of the region of interest
Filters	No filter, exponential, gradient, logarithm, square, square-root, local binary pattern

### Statistical analysis

We used the first cohort (n = 30) and second cohort (n = 68) as the training and validation datasets, respectively. The goal was to build a single *radiomic signature*, which is both robust to inter-reader reproducibility and prognostic of OS across the cohorts and readers. Constructing a single radiomic signature instead of using a set of features reduces feature space dimension mitigating multiple testing problem. In addition, a multi-feature signature accounts for the inter-feature interactions which usually leads to improved predictive modeling compared to individual features^10,26^.

First, individual radiomic features of both Reader 1 and 2 in the training set were evaluated for their inter-reader reproducibility by calculating Intraclass Correlation Coefficients (ICC) for each pair of features. ICC, which represents how strongly the features in the same class resemble each other, is generally regarded as poor if less than 0.3^27–29^. We excluded features with ICC < 0.3 to eliminate unstable features among the contours of both readers. The more reproducible features were then tested for their ability to predict OS in the training cohort using a Cox proportional-hazards regression model^30^ where the features were treated as continuous variables. This was done using Reader 1 contours as Reader 1 was the more experienced radiologist. Any features that were not prognostic of OS in the training cohort were eliminated. A feature selection method (LASSO^31^) was applied to significant features (P < 0.05) in the training cohort to select the ones with best prognostic power.

Each radiomic feature derived above in the training cohort was then tested in the validation cohort. This was done by retesting these selected features on both Reader 1 and 2 contours in the validation cohort using a univariate Cox regression model and Wald test. Given that this was the validation phase, to control the multiple testing problem, false discovery rate (FDR) control was applied^32^. The feature was considered validated if its adjusted P-value was <0.05.

As a final step, the remaining significant features in the training cohort were run through a Cox regression model to generate a single radiomic signature. To validate the reproducibility of the constructed radiomic signature, the ICC was calculated in both training and validation cohorts. To validate the prognostic value of the constructed radiomic signature, Cox regression and Wald test were run in the validation cohort for both Reader 1 and 2 contours.

Clinical factors that may be prognostic of OS were also used in univariate Cox regression models. These factors include age, sex, tumour size, grade, N stage, margin, and CA 19-9 levels.

## Results

Out of 410 initial radiomic features generated from PyRadiomics library, 133 features were removed due to having zero or constant values for all patients. Out of 277 remaining features, 251 features had ICC > 0.3 among the contours of two readers in the training cohort. When the Cox regression model followed by feature selection was applied to these 251 reproducible features in the training cohort with Reader 1 contours, 3 features were significant (P < 0.05). These 3 features were then assessed using Cox regression in the validation cohort with both readers’ contours, and 2 remained significant after applying FDR multiple testing control (P < 0.05). Table 3 summarizes the hazard ratios, P-values, and ICC values for these significant radiomic features (and radiomic signature) for prognostication of OS in the training and validation cohorts. It also lists the median values of the significant features (and radiomic signature) in the training cohort, which were used for dichotomization of the validation cohort.

**Table 3:** List of hazard ratios, P-values, median values, and ICCs of significant radiomic features prognostic of OS in the training and validation cohorts.

Radiomic Feature	Hazard Ratio (HR) and P-value in Validation Cohort Reader 1	Hazard Ratio and P-value in Validation Cohort Reader 2	Median Value in Training Cohort	ICC in Training Cohort	ICC in Validation Cohort
Original_glcm_ SumEntropy	HR = 1.41 (CI: 1.04–1.92) P = 0.036	HR = 1.39 (CI: 1.05–1.84) P = 0.042	−0.05	0.33	0.72
squareroot_glcm_ClusterTendency	HR = 1.39 (CI: 1.04–1.86) P = 0.036	HR = 1.40 (CI: 1.04–1.88) P = 0.042	−0.33	0.70	0.63
Radiomic Signature	HR = 1.56 (CI: 1.15–2.13) P = 0.005	HR = 1.35 (CI: 1.04–1.75) P = 0.022	0.94	0.46	0.63

Abbreviations: CI: confidence interval; ICC: intraclass correlation; OS: overall survival: Original_glcm_SumEntropy, sum entropy feature extracted from original image via grey level co-occurrence matrix; Squareroot_glcm_ ClusterTendency: cluster tendency feature extracted from filtered image (square root) via grey level co-occurrence matrix.

**Figure 1:**
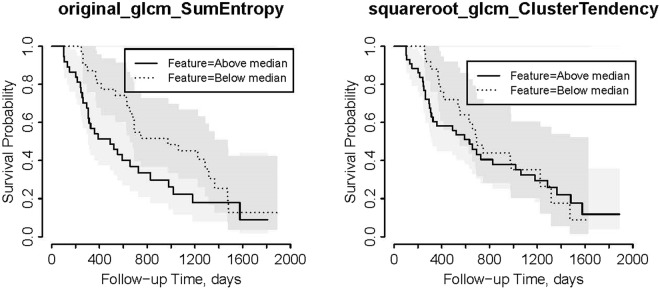
Kaplan-Meier plots for OS for the validation cohort with Reader 1 contours dichotomized based on the median values of significant features in the training cohort.

**Figure 2:**
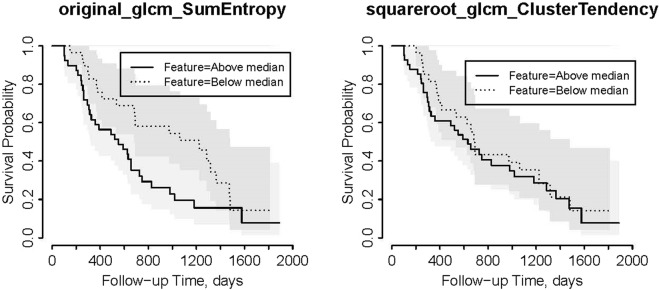
Kaplan-Meier plots for OS for the validation cohort with Reader 2 contours dichotomized based on the median values of significant features in the training cohort.

Figures 1 and 2 show the Kaplan-Meier plots of cumulative OS for these 2 radiomic features in the validation cohort for Reader 1 and 2, respectively.

As it can be seen from Table 3, the 2 significant features are second order features extracted from GLCM matrix: one feature is Sum Entropy calculated on the original image and the other is Cluster Tendency calculated on the filtered images (squared root). Sum Entropy is a texture feature that measures the randomness in the image as shown in Equation 1.

1 $${\rm{Sum}}\,{\rm{Entropy}}=-{\sum }_{k=2}^{2{N}_{g}}{P}_{x+y}(k)\mathrm{log}({P}_{x+y}(k))$$

where ${P}_{x+y}(k)={\sum }_{i=1}^{{N}_{g}}{\sum }_{j=1}^{{N}_{g}}P(i,j)where\,i+j=k=2,3,\ldots ,2{N}_{g}$ and P(i,j) is the probability of gray-level i to be adjacent to gray-level j in the image.

Cluster Tendency is a measure of groupings of pixels with similar gray-level values:

2 $${\rm{Cluster}}\,{\rm{Tendency}}={\sum }_{i=1}^{{N}_{g}}{\sum }_{j=1}^{{N}_{g}}{(i+j-{\mu }_{x}-{\mu }_{y})}^{2}P(i,j)$$

where *μ*_*x*_ and *μ*_*y*_ are the mean gray level intensities of the marginal row and column probabilities of *p*_*x*_ and *p*_*y*_, respectively where ${\mu }_{x}={\sum }_{i=1}^{{N}_{g}}{p}_{x}(i)i$, ${\mu }_{y}={\sum }_{j=1}^{{N}_{g}}{p}_{y}(j)j$, and ${p}_{x}(i)={\sum }_{j=1}^{{N}_{g}}P(i,j)$, ${p}_{y}(j)={\sum }_{i=1}^{{N}_{g}}P(i,j)$.

The radiomics signature derived from these 2 radiomic signatures combined is shown in Equation 3:

3 $${\rm{Radiomics}}\,{\rm{Signature}}={e}^{0.44\times {F}_{1}+0.11\times {F}_{2}}$$

where F_1_ is original_glcm_SumEntropy and F_2_ is squareroot_glcm_ClusterTendency.

The hazard ratios in the validation cohort for the radiomic signature were 1.56 (Confidence Interval (CI): 1.15–2.13) and 1.35 (CI: 1.04–1.75) for Reader 1 and 2, respectively. The P-values in the validation cohort for the radiomic signature were 0.005 and 0.022 for Reader 1 and Reader 2, respectively with ICC value of 0.63 (Table 3).

**Figure 3:**
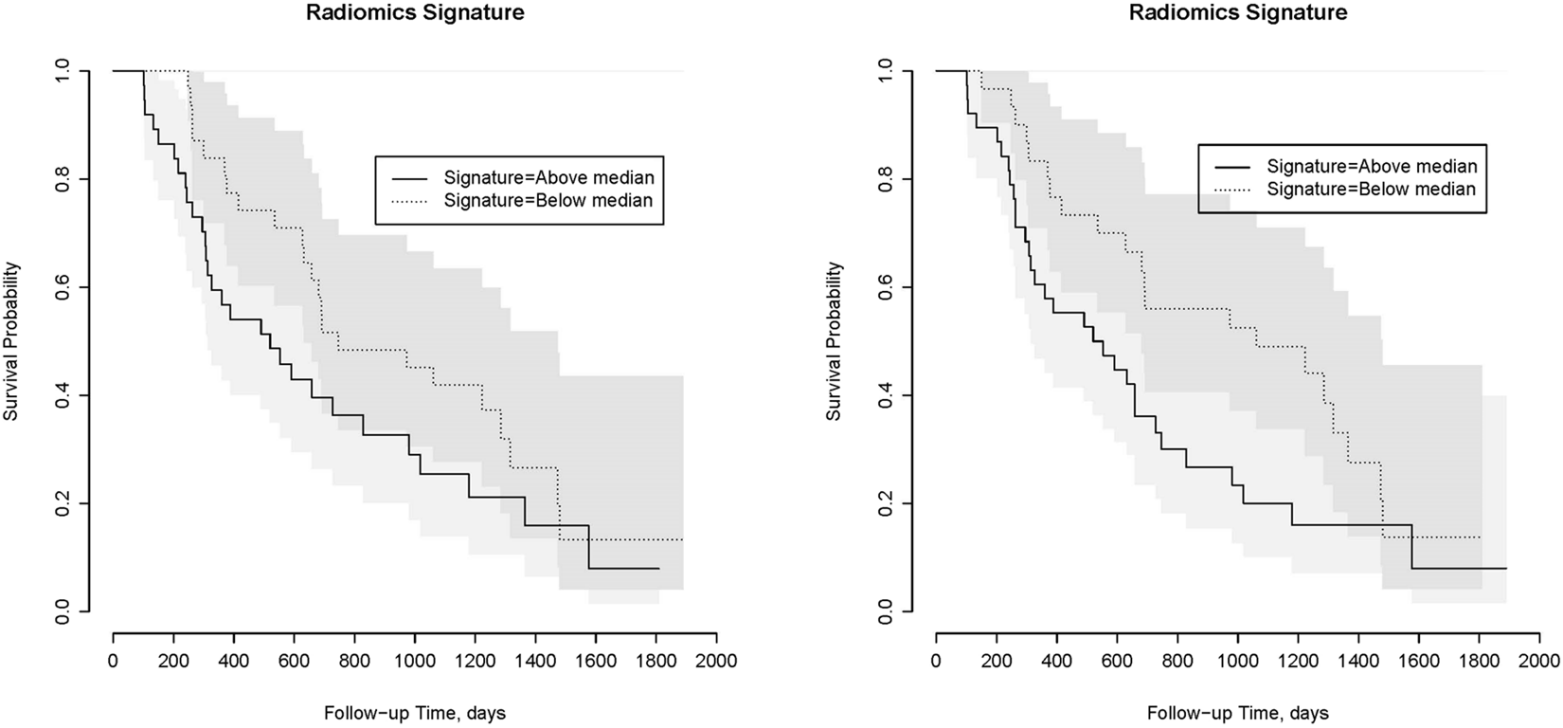
Kaplan-Meier plots for OS using Radiomic Signature. Left: Kaplan-Meier plots for OS for the validation cohort with Reader 1 contours dichotomized based on the median values of Radiomic Signature in the training cohort; hazard ratio: 1.56 (CI: 1.15–2.13), P-Value: 0.005, ICC: 0.63. Right: Kaplan-Meier plots for OS for the validation cohort with Reader 2 contours dichotomized based on the median values of Radiomic Signature in the training cohort; hazard ratio: 1.35 (CI: 1.04–1.75), P-Value = 0.022, ICC 0.63.

**Figure 4:**
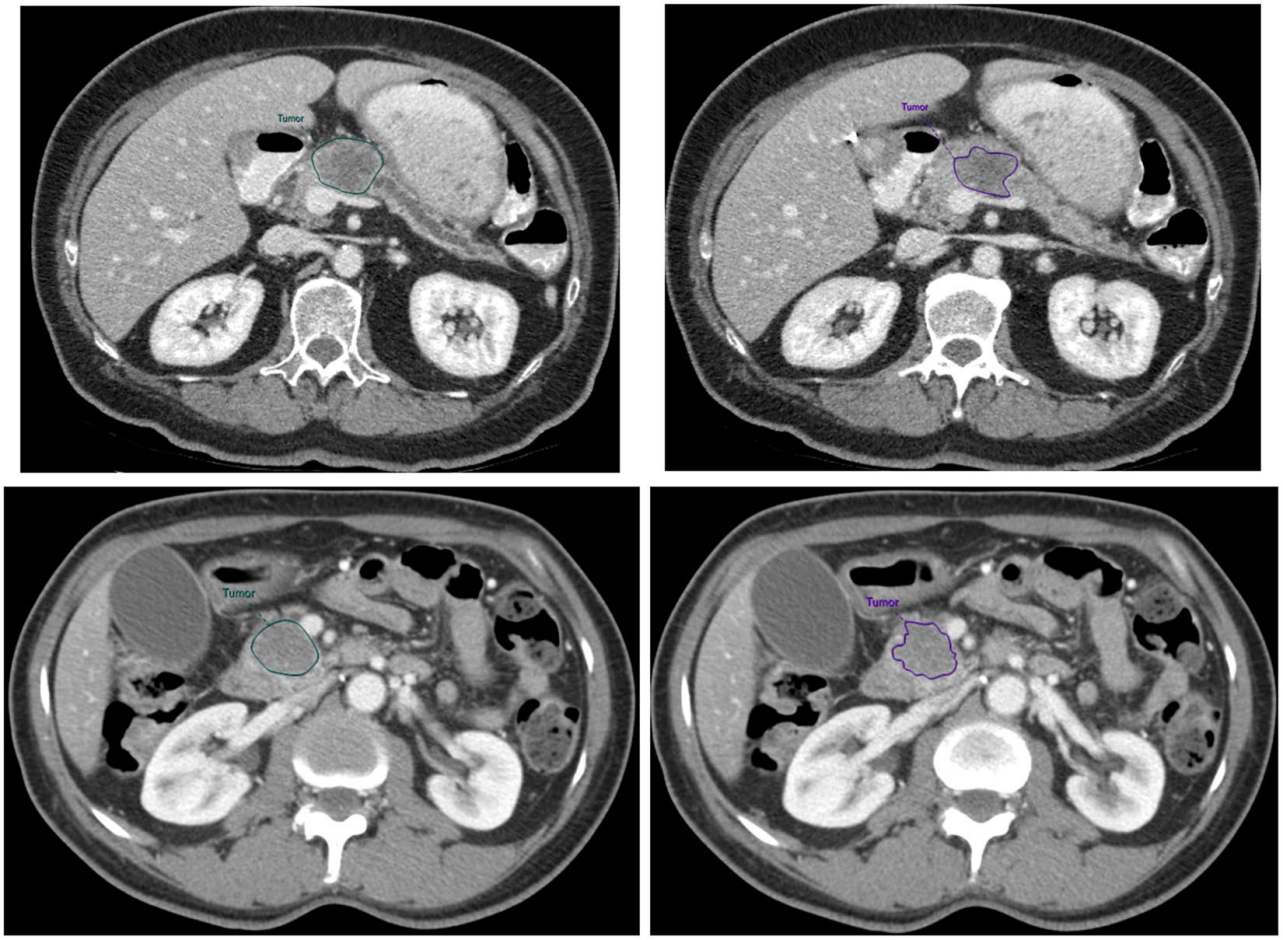
Representative patients from the validation cohort contoured for tumour with specific survival and radiomic signature values as follows: Left: Experienced Reader (Reader 1), Right: Inexperienced Reader (Reader 2). Top: Survival time: 4 months, Radiomic Signature - Reader 1: 1.25, Reader 2: 1.80. Bottom: Survival time: 44 months; Radiomic Signature - Reader 1: 0.49, Reader 2: 0.45.

Figure 3 shows the Kaplan-Meier plots for OS using the radiomics signature in the validation cohort for the two readers. Figure 4 shows two typical examples from the validation cohort contoured for tumour by both Reader 1 and 2 with specific survival time and radiomic signature values.

Out of clinical factors, only N stage was significant in the validation cohort with P-value of 0.03 and hazard ratio of 2.27 (CI: 1.06–4.86). To investigate whether the radiomic signature adds prognostic value to the model built with N stage, we tested a bivariate Cox regression model using N stage and radiomic signature and it was found that the bivariate model (N stage plus radiomic signature) is significantly different (with improved performance) than the univariate model (N stage alone) (Likelihood-ratio test P-value: 0.005). This indicates that adding radiomic signature to the clinical factor model (N stage) further improves the prognosis performance. CA19-9 levels were available for a subset of patients; 25 and 39 patients for training and validation cohorts, respectively. In the subset, CA19-9 factor was only significant in the validation cohort with P-value of 0.047 and hazard ratio of 1.37 (CI: 1.00-1.88). Adding radiomic signature to CA19-9 model significantly improved the prognostic performance of OS (P-value: 0.01) confirming that the radiomic signature adds to the prognostic power of the model with of CA19-9, which is an established clinical biomarker.

Table 4 summarizes the P-values for all clinical factors for prognostication of OS in the training and validation cohorts.

**Table 4:** List of P-values and hazard ratios for clinical factors for prognosis of OS in the training and validation cohorts.

Clinical Feature	Hazard Ratio (HR) and P-value in Training Cohort	Hazard Ratio (HR) and P-value in Validation Cohort
Age	HR = 1.01 (CI: 0.95–1.08) P = 0.69	HR = 1.02 (CI: 0.99–1.05) P = 0.22
Sex	HR = 0.95 (CI: 0.34–2.63) P = 0.92	HR = 0.93 (CI: 0.54–1.60) P = 0.78
Size	HR = 1.02 (CI: 0.60–1.75) P = 0.93	HR = 0.85 (CI: 0.69–1.14) P = 0.36
Grade (G2 vs. G1)	HR = 2.12 (CI: 0.27–17.46) P = 0.47	HR = 1.98 (CI: 0.98–4.01) P = 0.06
Grade (G3 vs. G1)	HR = 4.26 (CI: 0.50–36.03) P = 0.18	HR = 1.30 (CI: 0.43–3.87) P = 0.64
N Stage	HR = 0.42 (CI: 0.13–1.42) P = 0.16	HR = 2.27 (CI: 1.06–4.86) P = 0.03
Margin	HR = 0.47 (CI: 0.17–1.35) P = 0.16	HR = 1.17 (CI: 0.55–2.49) P = 0.69
CA19–9	HR = 1.15 (CI: 0.67–1.96) P = 0.61	HR = 1.37 (CI: 1.00–1.88) P = 0.047

Abbreviations: CI: confidence interval; OS: overall survival.

## Discussion

PDAC has a very low survival rate^33^. Better treatment options, fundamental understanding of the disease and earlier detection methods are needed. In this exploratory study, we evaluated the potential of radiomic features in PDAC on CT as part of early validation. We have demonstrated the potential of a radiomic signature as a prognostic biomarker in PDAC that can be used across different CT scanners and readers. Although radiomic features have been found to be prognostic of patient outcome in different cancer sites such as lung^7,18^, kidney^19,20^, and colorectal cancer^21^, there is limited work on PDAC^9,22,23^. These studies are all single institution exploring a limited number of radiomic features. In addition, only one reader contour has been used for the analysis, and standard radiomic libraries are not used in most of these studies. By using an open source code library (PyRadiomics^24^), there is an opportunity for other centres to validate the findings presented in this study. If further validated, this signature could be used to help select patients that may benefit from neoadjuvant treatment.

Radiomics studies for cancer prognosis are usually limited by the “Large P, small N” dataset problem^10^ where the number of features is far greater than the number of patients in the dataset. This challenge combined with the reproducibility issues inherent in different readers annotating the same image differently, and inconsistency in images acquired by different scanners, which might lead to unreliable features, cast doubt on the reproducibility of radiomic features as prognostic biomarkers for cancer. In this study, the main goal was to address these challenges by generating a single radiomic signature using the contours of two readers on two cohorts from two institutions where the first cohort was used for radiomic signature discovery and the second cohort was used for validation. This allowed us to separate the training and testing data and thus, to perform a proper validation of the generated radiomic signature.

Excluding features with low agreement between the readers ensured the reproducibility of the final radiomic signature. The radiomic signature was generated by combining the features that were significant in the training cohort and remained significant in the validation cohort after multiple testing correction. It is encouraging to observe that the radiomic signature that was generated in the training cohort remained significant in the validation cohort for both readers. This confirms the reproducibility of radiomic features as cancer biomarkers across not only different scanners/institutions which has also been shown in other studies for different cancer sites such as lung^34^ but also different readers.

It was interesting to observe that a significant number of radiomic features (251 out of 277) were robust with respect to inter-reader variability in ROI contouring. The fact that out of 277 robust features (with moderate and high ICC), only 3 were found to be prognostic of OS in the training cohort may be due to small sample size (n = 30). A larger sample size will increase the probability of finding more prognostic features in the training phase.

As an indicator of tumour heterogeneity, entropy-related radiomic features (e.g., entropy^9^, joint entropy^22^, and sum entropy^35^) have been shown to be prognostic of OS for different cancer sites. Entropy measures the degree of randomness or non-uniformity in the image and it has been hypothesized that it can act as a surrogate for tumour heterogeneity. The comparison of pairs of synchronous metastases from the same primary tumour has shown that entropy in each pair is highly correlated suggesting that it is capable of representing tumour biological characteristics^36^. It is promising to note that one of the radiomic features validated in this work is also based on entropy-related features (Sum Entropy), which strengthens the hypothesis that this specific radiomic feature may capture the underlying tumour phenotype.

Although tumour size measured as the maximal diameter of the mass on gross pathologic examination has been shown to be a histopathologic feature for prognosis of OS^5^, the corresponding radiomic feature (ROI diameter or ROI area) was not significant. This may be in part related to the poor definition of cancer margins and high interobserver variability in size measure on CT of PDAC. This indicates that features such as entropy that capture tumour characteristics beyond size may be needed for prognostication of PDAC.

Out of other clinical factors available for both cohorts (age, sex, tumour grade, N stage, and margin), only N stage, which is a postoperative factor was prognostic of OS in the validation cohort. It is important to note that the radiomic signature improved the prognostic power when added to N stage model. This indicates that the radiomic signature harbours prognostic information not necessarily captured by N stage factor. This, combined with the fact that radiomic signature is a preoperative biomarker, reconfirms the prognostic value of radiomic signature as a potentially reliable biomarker for PDAC. CA19-9 levels which had been shown to be associated with the OS of PDAC^37^ were available for a subset of patients in both cohorts and it was significant only in the validation cohort. Similar to N stage, the radiomic signature improved the prognostic power when combined with CA19-9.

Limitations of this work was the relatively small sample, and outcome was limited to overall survival. We hope to extend this work to larger cohorts and multicentre studies with more clinical outcome and genomics data soon. Moreover, CA19-9 and carcinoembryonic antigen (CEA) which have been shown to be associated with the OS of PDAC^37^ were not available for all patients. In future studies, the added prognostic value of radiomic signature to these preoperative biomarkers will be investigated using the full cohorts. Nevertheless, when these biomarkers are obtained, which is after diagnosis, radiomic features are readily available with no extra cost. Thus, a reliable radiomic signature with prognostic power is of significant value independent of other preoperative biomarkers.

Once validated, these biomarkers may have a role in selection of patients who should undergo neoadjuvant treatment with chemotherapy and/or radiation therapy prior to surgery. This also provides a potential signature to be tested in different PDAC populations such as unresectable patients. Further work on histologic correlates such as tumour stroma which is a potential druggable target in this disease would also be of interest.

In this study, we have demonstrated a set of imaging biomarkers and a signature that are both reproducible across different readers and CT scanners and prognostic in preoperative patients. These parameters provide a reasonable starting set of quantitative measures for prospective validation in future trials in surgical candidates with PDAC.

## Conclusion

Conventional staging CT-based radiomic features related to Sum Entropy and Cluster Tendency show promise for prognostication of OS for PDAC patients undergoing surgical resection across different institutions.

### Ethics approval and consent to participate

The Sunnybrook Health Sciences Centre and University Health Network Research Ethics Boards approved these retrospective single institution studies and waived the requirement for informed consent.

## Data Availability

The datasets generated and/or analyzed during the current study are available from the corresponding author on reasonable request pending the approval of the institution(s) and trial/study investigators who contributed to the dataset.
